# Targeting Steroid-Metabolizing Enzymes with 15β-Substituted Estrone Analogues: Dual Discovery of AKR1C2/17β-HSD1 Inhibitors and a Fluorescent 17β-HSD1 Ligand

**DOI:** 10.3390/cancers18121889

**Published:** 2026-06-10

**Authors:** Vivien Resch, Marija Gjorgoska, Eva Hafner, Ildikó Bacsa, Benjamin Kovács, Tomaž Büdefeld, Attila Hunyadi, Ildikó Huliák, Mónika Kiricsi, Gábor Paragi, Tea Lanišnik Rižner, Erzsébet Mernyák

**Affiliations:** 1 Department of Medicinal Chemistry, University of Szeged, Dóm tér 8, H-6720 Szeged, Hungaryparagi@sol.cc.u-szeged.hu (G.P.); 2 Institute of Biochemistry and Molecular Genetics, Faculty of Medicine, University of Ljubljana, Vrazov trg 2, 1000 Ljubljana, Slovenia; 3 Institute of Pharmacognosy, University of Szeged, Eötvös u. 6, H-6720 Szeged, Hungary; 4 HUN-REN-SZTE Biologically Active Natural Products Research Group, Eötvös u. 6, H-6720 Szeged, Hungary; 5 Department of Biochemistry and Molecular Biology, University of Szeged, Közép fasor 52, H-6726 Szeged, Hungary; 6 Institute of Physics, University of Pécs, H-7624 Pecs, Hungary; 7 Department of Theoretical Physics, University of Szeged, Tisza L. krt. 84-86, H-6720 Szeged, Hungary

**Keywords:** AKR1C1–3 enzymes, 17β-HSD1 enzyme, inhibitor, fluorescent labeling, chemoresistance, antiproliferative

## Abstract

Background/Objectives: Aldo–keto reductase isoforms AKR1C1–3 and 17β-hydroxysteroid dehydrogenase 1 and 2 (17β-HSD1 and 17β-HSD2) are key enzymes in steroid metabolism and validated targets in hormone-dependent cancers. Methods: In this study, Δ^15^- and 15β-substituted estrone derivatives were evaluated as inhibitors of AKR1C1–3 and 17β-HSD1 using enzymatic assays, cell viability assaysand computational modeling. Cellular uptake of the fluorescent estrone-based inhibitor was investigated using confocal microscopy. Results: The Δ^15^-estrone derivative showed potent and selective inhibition of 17β-HSD1 in the low nanomolar range, while 15β-*O*-propargyl and 15β-azide derivatives exhibited dual inhibitory activity against 17β-HSD1 and AKR1C2. The Δ^15^- and 15β-azide derivatives reduced cell viability in hormone-dependent breast, endometrial, and ovarian cancer cell lines in the sub- to low-micromolar range. A BODIPY-labeled 15β-*O*-propargyl analogue retained submicromolar inhibitory potency toward 17β-HSD1, representing the first fluorescent estrane-based inhibitor with preserved biological activity. Confocal microscopy confirmed efficient cellular uptake and predominant cytosolic localization in MCF-7 cells. Conclusions: These findings identify Δ^15^- and 15β-modified estrone derivatives as promising single- and dual-target inhibitors and introduce a fluorescent probe suitable for investigating intracellular steroid metabolism in hormone-dependent malignancies.

## Simple Summary

Hormone-dependent cancers such as breast, endometrial, and ovarian cancers often rely on enzymes that control estrogen production and activity, making these enzymes attractive targets for new treatments. This study aimed to evaluate structurally modified estrone derivatives that can block key enzymes involved in estrogen metabolism, with the goal of reducing cancer cell growth and improving target selectivity. The present findings identify D-ring-modified estrone derivatives as promising single- and dual-target inhibitors and introduce a fluorescent probe suitable for investigating intracellular steroid metabolism in hormone-dependent malignancies.

## 1. Introduction

Estrogens play essential roles in the pathogenesis of hormone-dependent malignancies, including breast, endometrial, and ovarian cancers (BC, EC, and OC, respectively). In addition to cancer, hormone-dependent disorders encompass several non-malignant conditions such as endometriosis and polycystic ovary syndrome, which can profoundly affect patients’ quality of life [1]. Owing to the central role of estrogens in promoting cell proliferation and their potential genotoxic effects, inhibition of estrogen signaling represents an established therapeutic strategy for estrogen-dependent malignancies as well as several non-oncologic conditions.

**Figure 1:**
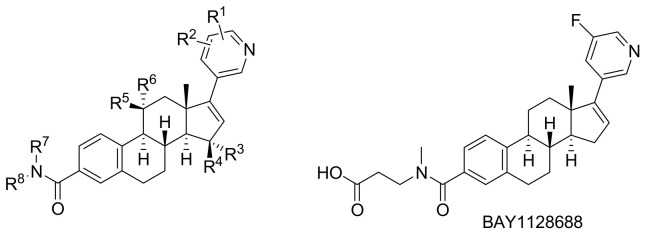
The general formula of patented AKR1C3 inhibitors and the most promising derivative.

Aldo-keto reductase enzymes AKR1C1, AKR1C2, and AKR1C3 display distinct catalytic preferences for 3-, 17-, and 20-ketosteroid substrates and are broadly expressed in peripheral tissues, where they regulate local steroid hormone availability and receptor occupancy [2]. Dysregulated AKR1C3 expression contributes to intratumoral androgen biosynthesis in breast cancer, castration-resistant prostate cancer, and polycystic ovary syndrome. Moreover, increasing evidence indicates that AKR1C enzymes mediate resistance to cancer chemotherapy, mainly via carbonyl reduction and metabolic inactivation of therapeutic agents [3]. Consequently, AKR1C isoforms have emerged as relevant drug targets for the treatment of steroid hormone-dependent cancers and endocrine disorders. The literature reveals several structurally different inhibitors of AKR1C1–3 enzymes, albeit selective inhibition of the three isoforms is rather challenging [2,4]. Especially that of AKR1C1 and AKR1C2 is difficult, as these enzymes differ by only one active site amino acid residue. Bayer Pharma has patented estra-1,3,5(10),16-tetraene-3 carboxamides as AKR1C3 inhibitors, including compounds having a C-15-substituent (US2016/0024142A1, Figure 1). One compound (BAY1128688) has been tested in a phase I and II clinical trial in endometriosis patients; however, the phase was terminated due to the hepatotoxicity of the derivative [5].

17β-HSD1 is one of the most studied enzymes involved in estrogen biosynthesis. The two main limitations concerning the development of 17β-HSD1 inhibitors are their estrogenic activity and the inhibitory activity against other 17β-HSD isoforms. For all these reasons, to date, only one 17β-HSD1 inhibitor, an E1 derivative bearing a 15β-substituent developed by Organon Finland (OG-6219, Linustedastat), has reached the clinical trial stage for endometriosis (https://www.elenaendometriosisstudy.com/#!/, accessed on 15 April 2026). Messinger’s group [6] and Schneider’s group [7] developed an additional series of 15β-substituted E1-based 17β-HSD1 inhibitors, some of which exhibited IC_50_ values in the nanomolar range.

Fluorescent inhibitors constitute an effective strategy for developing and characterizing enzyme inhibitors [8,9]. Owing to safety and regulatory considerations, fluorescence-based assays are increasingly replacing radioactivity-based approaches in biochemical and cellular studies. Poirier’s group recently reported the first fluorescently labeled steroidal inhibitors of 17β-HSD3 or STS, generated by conjugation of the steroid to a dansyl moiety [10,11]. Confocal microscopy studies demonstrated efficient cellular uptake of these probes and their localization within specific intracellular compartments. To the best of our knowledge, however, fluorescently labeled steroidal inhibitors targeting 17β-HSD1 or AKR1C1–3 have not yet been described.

Despite substantial effort, progress in developing inhibitors of 17β-HSD1 and AKR1C1–3 has been limited, and only a few inhibitors have advanced to clinical evaluation. This highlights an unmet need for next-generation agents with improved potency, selectivity, and tolerability.

**Figure 2:**
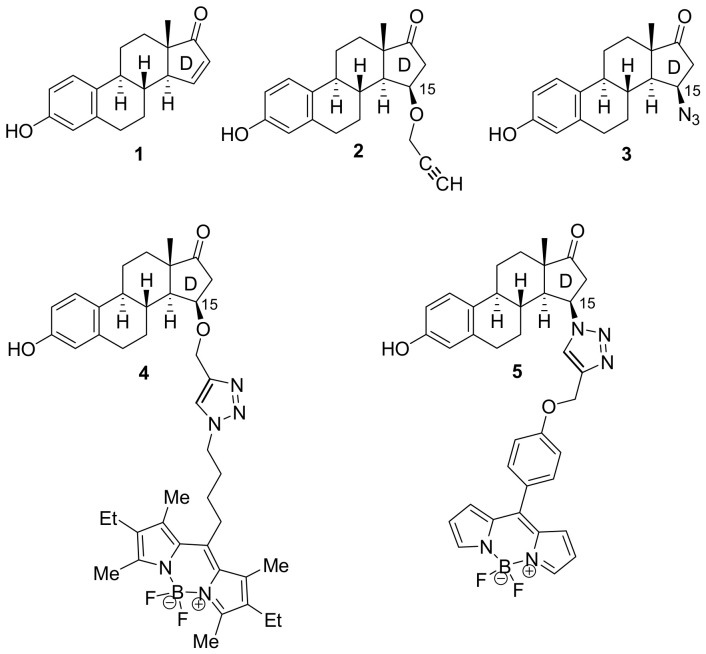
Structures of test compounds **1**–**5**.

With these considerations in mind, our objective was to perform a comparative in vitro evaluation of the inhibitory activities of the two previously synthesized BODIPY-labeled estrone derivatives (**4** and **5**) and their non-fluorescent precursors (**1**–**3**, Figure 2 [12]) against 17β-HSD1, 17β-HSD2, and AKR1C1–3. In parallel, computational analyses, including molecular docking and molecular dynamics simulations, were performed to elucidate the molecular determinants of enzyme binding and selectivity. A further aim of this study was to evaluate the potential antiproliferative effect of the compounds against MCF-7 breast, endometrial (KLE, HEC-1-A, Ishikawa, and RL-95), and ovarian (COV362, Kuramochi, and OVSAHO) cancer cell lines and their corresponding three control cell lines (MCF10A, HIEEC, and HIO80).

## 2. Results

### 2.1. Enzyme Inhibition Studies

We previously reported the synthesis of 15β-substituted, fluorescently labeled estrone derivatives (compounds **4** and **5**, Figure 2, [12]). The conjugates were obtained via Cu(I)-catalyzed azide–alkyne cycloaddition, employing 4,4-difluoro-4-bora-3a,4a-diaza-s-indacene (BODIPY) fluorophores as labels (Figure 2). The two 15β-substituted, fluorescently labeled estrone derivatives (**4** and **5**) and their precursors (**1**–**3**) were here evaluated as inhibitors of AKR1C1–3, 17β-HSD1, and 17β-HSD2 enzymes. The compounds were tested against AKR1C1–3 enzymes, using 1-acenaphthenol as a substrate (Table 1). Compounds **1** and **2** at a 100 µM test concentration showed <33% inhibition of isoenzyme AKR1C1, but the azide (**3**) displayed an inhibition % of 62.5. However, the 15β-*O*-propargyl (**2**) and azide (**3**) derivatives showed strong inhibition of isoenzyme AKR1C2, with IC_50_ values of 0.67 µM and 4.27 µM, respectively. No inhibition was seen for the three precursors **1**–**3** on AKR1C3, except for the *O*-propargyl derivative (**2**), but only at the highest test concentration (56.2% inhibition at 100 µM). The labeled derivatives (**4** and **5**) displayed very weak inhibition (≤20% at 20 µM) of AKR1C1–3 enzymes.

The initial screening of precursors **1**–**3** at 100 µM against 17β-HSD1 demonstrated pronounced inhibitory effects. Encouraged by these results, dose–response studies were performed to determine IC_50_ values, which indicated remarkable potency, as all compounds displayed IC_50_ values in the low nanomolar range (<5 nM). Nevertheless, the two labeled derivatives **4** and **5** showed a marked difference at a 20 µM test concentration. Based on their inhibition % data, the IC_50_ value was determined for compound **4**, falling in the submicromolar range (0.9 µM).

Compounds **1** and **3** exerted moderate 17β-HSD2 inhibitory activity, with inhibition% data slightly above 70% at a 100 µM test concentration. The other test compounds (**2**, **4**, and **5**) did not exhibit substantial inhibition of the 17β-HSD2 enzyme.

### 2.2. Cellular Assays

The antiproliferative effects of the compounds (**1**–**5**) were investigated on different cancer (MCF-7, KLE, HEC-1-A, ISHIKAWA, RL-95, COV362, KURAMOCHI, OVSAHO) and control cell lines (MCF10A, HIEEC and HIO80). At first, cell viability was assessed at a 100 μM test concentration (Table 2). Overall, precursors **1** and **3** exhibited strong growth-inhibitory effects across seven of the investigated cell lines, including both tumor and nontumor control cell lines, although their activity profiles differed among the individual cell types. The 15β-*O*-propargyl derivative (**2**) proved to be potent against four endometrial cell lines; however, its potency was lower compared to compounds **1** and **3**. In contrast, the fluorescently labeled derivatives (**4** and **5**) did not exhibit marked antiproliferative activity in any of the tested cell lines. Based on the preliminary screening, EC_50_ values were determined for the most potent compounds (**1**–**3**). The α,β-unsaturated ketone (**1**) displayed substantial antiproliferative activity against nine cell lines, including the three control cell lines. The lowest submicromolar EC_50_ value was observed on the MCF10A control cell line; however, all calculated EC_50_ values remained within the low micromolar range. Regarding the EC, at least a twofold selectivity was observed compared to the control cell line, with higher potency against cancer cells. A similar tendency was observed for compound **3** on EC. In contrast, compounds **1** and **3** exhibited a more pronounced antiproliferative effect on the ovarian control cell line than on OC cells. Compound **2** displayed higher EC_50_ values, exceeding 10 μM across the tested cell lines.

**Figure 3:**
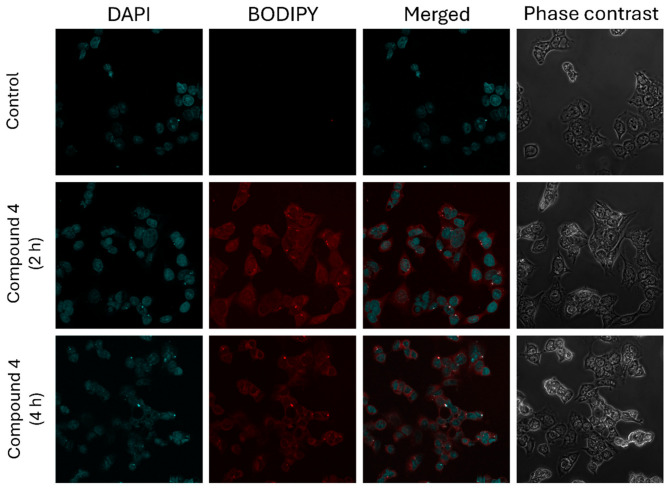
Confocal fluorescent microscopic images of MCF-7 cells incubated in the presence or absence of 10 µM compound **4**. The fluorescent signal indicates massive cytosolic accumulation of the molecule. Images were taken after 2 and 4 h treatments, and nuclei were stained with DAPI. The scale bar represents 20 µm.

Fluorescent confocal microscopy experiments were performed on MCF-7 breast adenocarcinoma cells to examine the uptake and intracellular localization of compound **4** (Figure 3). After both 2 and 4 h treatments of MCF-7 cells with 10 µM of the test compound, the compound-derived fluorescence was observed mainly in the cytosol and around the cell nuclei. The results clearly indicate that compound **4** is efficiently taken up by MCF-7 cancerous cells.

### 2.3. Computational Simulations

**Figure 4:**
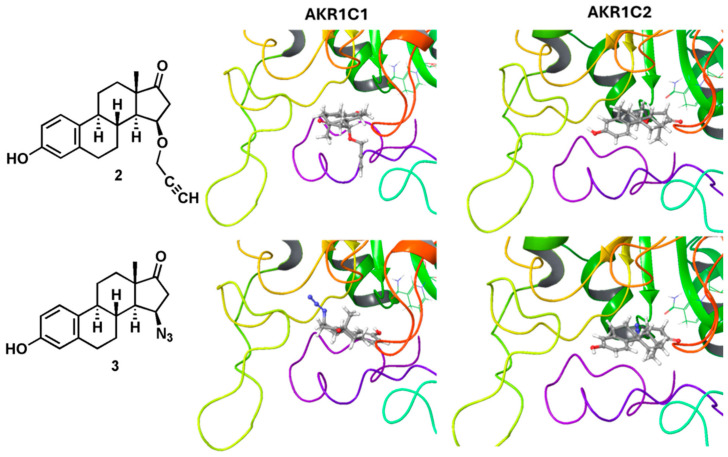
The best docking pose of ligands 2 and 3 in the AKR1C1 and AKR1C2 enzymes.

First, molecular docking calculations and interaction analysis were performed to gain a molecular-level insight into the possible binding mode of the most potent AKR1C2 inhibitors **2** and **3** in the active sites of AKR1C1 and AKR1C2 enzymes. To evaluate the reliability of the predicted binding modes, docking scores (Glide scores) were compared to the binding free-energy values derived from the experimental inhibition data. As Table 3 shows, similar tendencies can be observed between the theoretical and experimental data for both receptors (see more in Table S1). Moreover, the re-docking pose of the crystal steroid ligand closely matched the experimental geometry, further supporting the reliability of the applied docking protocol. More precisely, in the case of AKR1C1, the RMSD value is 0.248 Å, and in the case of AKR1C2, the value is 0.476 Å. Concerning the docking geometries of the modified steroids, the substituted D-ring is oriented outwards from the NADPH cofactor, facing the bulk water in the AKR1C1–ligand complexes, whereas in the AKR1C2–ligand complexes, this ring is located towards the cofactor, as illustrated in Figure 4.

**Figure 5:**
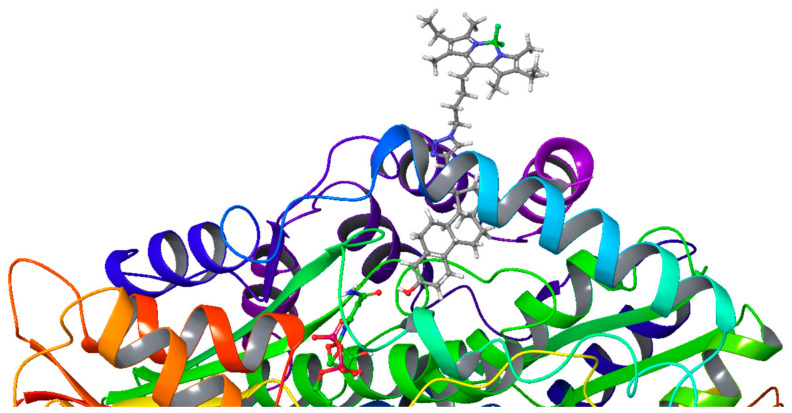
Starting structure of the ligand **4** and 17β-HSD1 enzyme complex.

Regarding the **4**–17β-HSD1 complex, the structural stability was investigated by molecular dynamics simulations. The ligand stability was illustrated by RMFS diagrams (Supporting Information, Figure S1), where the starting structure (Figure 5) was created according to the process described in the Materials and Methods Section (Section 4.3). We found that in all three independent simulations, the steroid part remained stable in the binding pocket, which suggests the modified steroid binding mode concerning the fluorescent conjugate.

## 3. Discussion

We previously synthesized the C-15-labeled BODIPY–estrone conjugates **4** and **5**, using CuAAC as the key transformation. Given the extensive use of BODIPY fluorophores in biomedical imaging [13], these conjugates may serve as valuable molecular probes for investigating interactions with enzymes involved in estrogen biosynthesis and metabolism, as well as with estrogen receptors (ERs). Moreover, considering the structural and functional differences among the aforementioned proteins, the 15β-labeled fluorescent estrone derivatives **4** and **5** may exhibit differential utility across biological applications.

Estrone derivatives functionalized at position C-15 possess diminished estrogenic or antiestrogenic activity due to the low ER-binding affinity of the compounds [14]. The steric constraints of the ER at the C-15 position of the ligand limit the accommodation of bulky substituents, leading to significantly reduced binding of C-15-substituted derivatives.

The reduced estrogenic activity of C-15-substituted estrone derivatives enables the development of potent inhibitors based on this scaffold, targeting enzymes involved in estrogen biosynthesis and metabolism. Assessing the selective inhibitory potential of these candidates is particularly critical. Careful evaluation of each member within a given enzyme family is essential, especially for enzymes with structurally similar active sites. Equally important is the comparative analysis of inhibitors for enzymes catalyzing reversible reactions. Such comprehensive studies may help prevent the clinical failure of otherwise potent inhibitors due to unforeseen toxicity or other side effects.

The results obtained here for compounds **1**–**5** on AKR1C1–3 enzymes reveal that the modification pattern of the D-ring greatly influences the inhibitory behavior. The presence of relatively small 15β-substituents (in compounds **2** and **3**) markedly enhances the inhibitory activity on the AKR1C2 isoenzyme. These inhibitors demonstrated strong potency on the two structurally more similar AKRs. In contrast, the fluorescent-labeled compounds **4** and **5** showed negligible inhibition on all tested AKRs, likely due to the steric impact of the bulky C-15 substituent.

**Figure 6:**
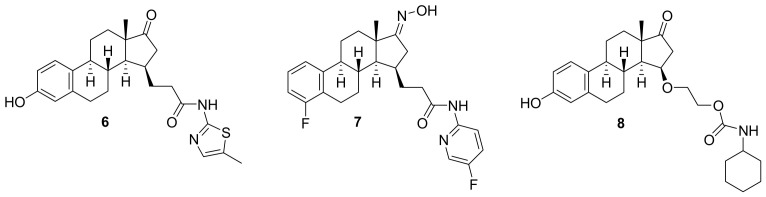
Potent estrone-based C-15-substituted 17β-HSD1 inhibitors.

Evaluation of the inhibitory activities of compounds **1**–**5** against 17β-HSD1 demonstrated that all members of the series exhibited substantial potency, with the sole exception of compound **5**, which contains a triazole ring directly attached to C-15 in the β-orientation. The pronounced inhibitory effects of the 15β-substituted derivatives **2** and **3** are consistent with previously reported structure–activity relationships [6,7]. Figure 6 highlights certain potent 15β-substituted nano- or submicromolar 17β-HSD1 inhibitors (**6**–**8**). The first two inhibitors **6** and **7** (OG-6219, Linustedastat) share a common structural motif: a 15β-positioned substituent connected via a C–C bond and a two-carbon alkyl linker. In contrast, the inhibitor developed by Schneider’s group (**8**) is a 15β-oxa-linked derivative [7].

Messinger et al. provided an explanation for the enhanced affinity of 15-substituted estrone derivatives for the 17β-HSD1 enzyme [6]. There is an opening towards the environment at the C-15-position of the ligand; a hole where the position of certain amino acids is highly flexible. SAR studies described in the literature show that establishment of both C–C and C–O bonds in 15β-orientation might be beneficial concerning the approval of inhibitory potential of the unsubstituted counterpart. The flexibility of the enzyme binding site likely enables both 15-carbo and 15-oxa derivatives with different stereochemistry to exhibit favorable binding characteristics. These results encouraged us to utilize the C-15-substitution strategy in the development of BODIPY-labeled estrone derivatives as inhibitors of 17β-HSD1. To our great delight, our targeted strategy proved successful. Notably, one BODIPY–estrone conjugate, **4**, retained high affinity, displaying submicromolar inhibition. The short linker (15-*O*-CH_2_) present in compound **4** may confer a favorable spatial arrangement within the binding pocket. In agreement with the findings of Messinger et al., who identified a preference for inhibitors with short (CH_2_-CH_2_) substituent chains, we assume that compound **4** might accommodate conveniently in the binding pocket. The presence of a freely rotatable four-carbon-long alkyl linker at the *meso*-position of the BODIPY core might also be a further advantage. The reduced affinity of conjugate **5** for the enzyme may be attributed to the increased rigidity of its C-15 substituent, in which two aromatic rings (triazole and phenyl) are connected via a short linker.

We investigated the antiproliferative effect of the test compounds (**1**–**5**) on hormone-dependent BC, EC, and OC cell lines. In order to investigate the effects of estrogens on tumor development, it is essential to understand their biosynthetic and metabolic pathways within the given tumor. Estrogens exert a dual effect in the development and progression of estrogen-dependent cancers. Through ERs, estrogens enhance gene expression and drive cell proliferation. However, oxidative metabolites formed from estrogens can induce DNA damage and trigger neoplastic transformation [15,16]. The impact of estrogens may differ according to the nature and grade of the tumor [17]. Estrogens exert their effects via ERα or ERβ nuclear and/or GPER membrane receptors. ERα activation promotes cell proliferation, while ERβ acts as a tumor suppressor [18]. E2 enhances the proliferation of grade 1 Ishikawa EC cells and grade 2 RL95–2 cells. E1 and E1S likewise stimulate the proliferation of Ishikawa cells and tend to increase the proliferation of HEC-1-A and RL95–2 cells while reducing the proliferation of KLE cells [17]. The observed differences in proliferative responses are linked to ERα positivity in Ishikawa cells and to GPER expression in other cells. The literature reveals that HIEEC, Ishikawa, and HEC-1A cells are capable of estrogen formation exclusively via the sulfatase pathway [19]. The EC cell lines under investigation exhibit differences in 17β-HSD1 and 17β-HSD2 expression levels, which may affect their responsiveness to antiproliferative compounds. Additionally, these cell types express all major genes involved in the production of hydroxyestrogens and estrogen quinones, as well as in their conjugation.

Concerning the OC models, distinct cell lines exhibit variability in E2 formation, and these gene expression differences are linked to chemoresistance [20]. The investigated OC models display increasing chemoresistance in the following order: OVSAHO < Kuramochi < COV362 [20]. The literature data indicate high 17β-HSD1 expression in OVSAHO cells, whereas it is undetectable in COV362 cells; conversely, 17β-HSD2 expression is high in COV362 cells but absent in OVSAHO cells. Accordingly, the variability in E2 formation of these OC cell lines may greatly influence their estrogen dependence and response to antiproliferative agents. Estrogens are known to play a crucial role in OVSAHO cell biology, while chemoresistance in these models is strongly linked to their gene expression profiles [21].

Compounds that influence one or multiple steps of estrogen biosynthesis and metabolism may exert a significant impact on tumor cell proliferation. The present study identified certain potent antiproliferative compounds active against different types of hormone-dependent cancers. Among them, compound **1** proved to be one of the most potent derivatives, as it inhibited the growth of the highest number of investigated cell lines (nine). This estrone-based derivative belongs to the series of α,β-unsaturated ketones, which may function as Michael acceptors. Such compounds can potentially undergo nucleophilic attack by amino acid residues of cancer-related proteins, as reported previously [22]. Accordingly, compound **1** may exert a dual mode of action: in addition to its inhibitory effect on 17β-HSD1, its Michael acceptor character may contribute to the pronounced antiproliferative activity observed. In estrogen receptor-positive MCF-7 breast cancer cells, compound **1** exhibited high antiproliferative potency accompanied by low selectivity toward the control breast cell line (approximately eightfold). In contrast, this selectivity pattern was reversed in endometrial cancer (EC) cell lines compared to their control, albeit to a lesser extent (approximately twofold). Concerning the 17β-HSD1 expression levels: higher expression of 17β-HSD1 has been reported in KLE, Ishikawa, and HEC-1-A cells compared to HIEEC and RL-95 cells, which may partly account for the observed differences in antiproliferative responses [19]. Thus, the potent 17β-HSD1 inhibitory activity of compound **1** may contribute to reduced survival of cell lines characterized by elevated enzyme expression. In contrast, the growth of RL-95 cells, which exhibit high 17β-HSD2 expression, was not markedly affected by any of the test compounds. Saturation of the Δ^15^ double bond (resulting in the loss of Michael acceptor character) in compounds **2** and **3** led to altered antiproliferative activity. Although the 15-azido derivative (**3**) displayed EC_50_ values comparable to those of compound **1** in seven cell lines, the high potency against HEC-1-A and COV362 cells was no longer observed. Regarding OC cell lines, none of the compounds showed significant activity against OVSAHO cells. Compound **1** demonstrated notable potency against both COV362 and Kuramochi cell lines, whereas compound **3** inhibited cell growth only in Kuramochi cells. Notably, the growth of the most chemoresistant COV362 cell line was inhibited exclusively by compound **1**. The 15-*O*-propargyl derivative (**2**) exhibited lower antiproliferative potency compared to compounds **1** and **3**; however, it showed selectivity toward EC cell lines. Fluorescent labeling of compounds **2** and **3** resulted in reduced antiproliferative activity. Although compound **4** retained its 17β-HSD1 inhibitory potency, conjugation with a BODIPY dye caused a substantial decrease in its antiproliferative potential. The relationship between the AKR1C2 inhibitory activity and the antiproliferative effects of compounds **2** and **3** in the studied cell lines warrants further investigation.Further compound synthesis and comprehensive evaluation are required to clarify the mechanisms of antiproliferative action and their relationship to compound structure and cell line gene expression profiles.

Wild-type MCF-7 cells exhibit low endogenous expression of 17β-HSD1, with the enzyme predominantly localized in the cytosol [23,24]. Accordingly, transfected MCF-7 models are commonly applied to study the functional consequences of 17β-HSD1 overexpression, including altered protein expression patterns and E2 formation. In contrast, the present study aimed to assess the cellular uptake and subcellular distribution of the BODIPY-labeled 17β-HSD1 inhibitor 4; therefore, wild-type MCF-7 cells were considered suitable for these investigations. Fluorescent confocal microscopy was employed to monitor the intracellular localization of compound **4**, utilizing the emission of the BODIPY fluorophore at approximately 580 nm. Untreated control cells were included to exclude significant cellular autofluorescence under the applied imaging conditions. Given the cytosolic localization of 17β-HSD1, these experiments were specifically designed to determine whether the fluorescently labeled E1 derivative can access the intracellular compartment required for potential target interaction. Although inhibitory activity was established in a recombinant enzyme system, the present imaging experiments were not intended to demonstrate intracellular enzyme inhibition or direct target engagement, but rather to verify cellular permeability and intracellular distribution. The results demonstrate efficient cellular uptake of compound **4** and its predominant accumulation in the cytosol of MCF-7 cells. Overlay images (Figure 3) revealed a diffuse cytoplasmic distribution surrounding the DAPI-stained nuclei, with no indication of nuclear localization. This distribution pattern is consistent with the reported intracellular localization of 17β-HSD1 and suggests that the compound reaches the relevant subcellular compartment. Taken together, these findings provide experimental evidence for the cellular accessibility of the inhibitor and support its utility as a preliminary fluorescent tool compound for studying intracellular steroid-metabolism-related processes. However, additional studies, including colocalization experiments, live-cell imaging, and competitive target-engagement assays, will be required to further validate its applicability as a dedicated imaging probe.

**Figure 7:**
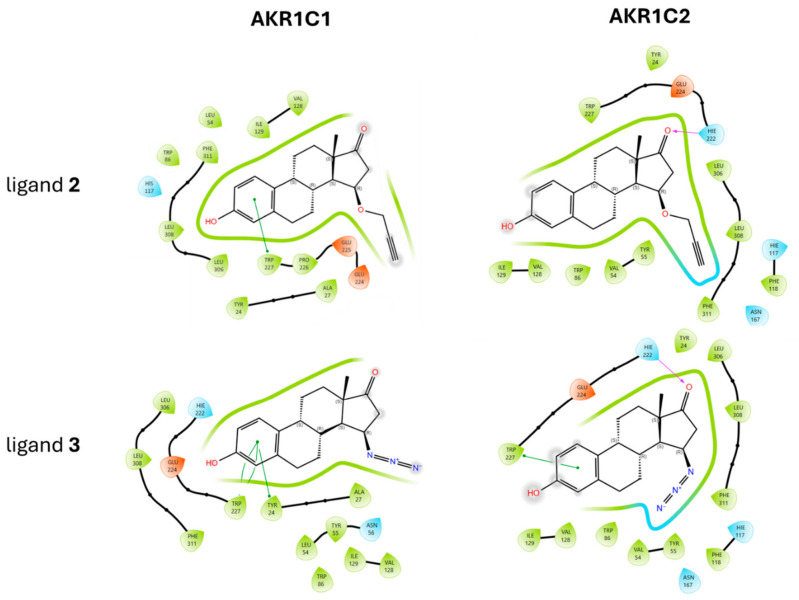
Ligand Interaction Diagrams (LIDs) of the AKR–ligand complexes.

Based on the inhibition data obtained for test compounds **1**–**5** against AKR1C1–3 enzymes, the most potent AKR1C2 inhibitors (compounds **2** and **3**) were selected for docking calculations. A comparative study involving AKR1C1 and AKR1C2 was performed to gain insight into the markedly different inhibitory activities of these compounds toward the two structurally highly similar enzymes. The docking results indicated that both ligands exhibit stronger binding affinity for the AKR1C2 isoenzyme compared with AKR1C1 (cf. Table 2). This trend is consistent with the experimental inhibition data, where both compounds showed higher inhibition against AKR1C2 (approximately 97–99%) than against AKR1C1 (approximately 32–63%). Analysis of the binding poses revealed that the principal difference between the ligand–protein complexes arises from distinct ligand orientations. In AKR1C1, both ligands preferentially adopt a binding mode in which the A-ring is oriented toward the NADPH cofactor. In contrast, in AKR1C2, the ligands favor an orientation in which the sterane skeleton positions the D-ring in proximity to NADPH. These findings suggest that subtle structural differences between the two enzymes significantly influence ligand orientation and stability within the active site. In particular, the residue at position 54 (Leu54 in AKR1C1 vs. Val54 in AKR1C2) appears to modulate the hydrophobic and steric environment of the binding pocket, thereby contributing to more favorable ligand binding in AKR1C2. Ligand Interaction Diagram (LID) analysis further indicates that this difference may be associated with a hydrogen bond between the ligand keto group and HIE222 in AKR1C2, which compensates for or outweighs the π–π stacking interaction observed between the ligands and TRP227 in AKR1C1 (Figure 7). Overall, these results are consistent with the observed selectivity of compounds **2** and **3** toward AKR1C2 and highlight the importance of subtle active site variations in determining isoenzyme selectivity.

Following the AKR1C1 and AKR1C2 investigations, computational simulations were carried out to obtain information on the binding mode of the fluorescent conjugate **4**, which exhibits high retained submicromolar inhibitory potential. For the complex of conjugate **4** with the 17β-HSD1 enzyme, the RMSF plots (see SI) demonstrate a stable binding mode of the ligand with respect to the protein. Moreover, the RMSF curves clearly show that the 15β-substituent is more flexible than the steroid core, owing to the relatively fixed binding mode of the sterane skeleton within the binding pocket, whereas the BODIPY moiety is located entirely outside the pocket. The presence of the freely rotatable four-carbon-long linker at the *meso*-position of the BODIPY core likely enables more favorable and effective interactions within the active site. Furthermore, the BODIPY part interacts with several amino acid residues of the α-helix in the 17β-HSD1 enzyme (chain-A: F226-R281). These interactions consist predominantly of hydrophobic contacts and water-mediated bridges, the latter arising from the fact that the BODIPY moiety is largely solvent-exposed (see more in Figure S2 Simulation Interaction Diagrams). It should be emphasized that strong interactions with the solvent can, in certain cases, promote enhanced dissociation of the protein–ligand complex; however, the MD simulations did not indicate such an effect. Overall, our results suggest that the fixed binding mode of the steroid core within the binding pocket may account for the potent inhibitory activity of the conjugate, despite the external positioning of the BODIPY moiety.

## 4. Materials and Methods

### 4.1. Chemical Synthesis

Estrone derivatives **1**–**5** were synthesized as described elsewhere [12].

### 4.2. Enzymatic Assays

#### 4.2.1. Inhibition Assay for AKR1C1–3 Enzymes

The AKR1C1–3 recombinant enzymes were prepared as described previously [25]. The in vitro enzymatic activities of AKR1C1–3 were determined spectrophotometrically by monitoring the change in absorbance at 340 nm, indicative of NAD^+^ reduction to NADH (ε_λ340_ = 6220 M^−1^ cm^−1^), in the presence of the substrate 1-acenaphthenol. The assays were performed in 300 µL reaction mixtures in 100 mM potassium phosphate buffer (pH 9.0), 0.005% (*v*/*v*) Triton X-114, 0.05% (*v*/*v*) DMSO, 2.3 mM NAD^+^, and 1-acenaphthenol (final substrate concentrations: 30 µM for AKR1C1, 60.0 µM for AKR1C2, and 100 µM for AKR1C3; [26]) (all Sigma Aldrich, Taufkirchen, Germany). Five microliters of each tested compound in DMSO or DMSO (control) was added to the reaction mixture. The plates were incubated for 5 min at 37 °C, and then background absorbance at 340 nm was measured using a microplate reader (PowerWave XS, Biotek, Winooski, VT, USA). The reactions were then started by the addition of 15 microliters of the enzyme, at final concentrations of 0.1 µM for AKR1C1, 0.3 µM for AKR1C2, and 1.5 µM for AKR1C3 (diluted in PBS; #D5652, Sigma Aldrich, Taufkirchen, Germany). Absorbance at 340 nm was measured at 8 s intervals over a 3 min period.

The measurements were performed in duplicate and were repeated as two independent experiments. Data analysis involved background correction followed by calculation of the slope (rate of NADH formation). Residual enzyme activity in the presence of inhibitors was expressed relative to the activity observed in the control (DMSO). The IC_50_ values were determined from the plots of residual activity versus log_10_ (inhibitor concentration) using GraphPad Prism, version 10.0 (GraphPad Software, Inc., San Diego, CA, USA).

#### 4.2.2. Inhibition Assay for 17β-HSD1

The 17β-HSD1 enzymatic assay with ^3^H-labeled estrone was performed as follows:

The bacterial homogenate of E. coli overexpressing 17β-HSD1 was previously prepared as described [27,28,29] and stored in aliquots at −80 °C. Then, 5 µL bacterial homogenate was added to 300 µL reaction mixtures containing 100 mM sodium phosphate buffer (pH 6.5), cofactor NADPH (100 µM) (#AE14, Karl Roth, Karlsruhe, Germany), substrate E1 (#E2300-000, Steraloids, Newport, RI, USA) in DMSO (55 nM), tritium-labeled E1 ([2,4,6,7−^3^H-E1]; #NET319250UC, Perkin Elmer, Waltham, MA, USA) (0.6 µCi/mL), and 10 µL of test inhibitor in DMSO (or DMSO for controls; final DMSO concentration 5%). The reactions were incubated in a water bath at 37 °C for 10 min (≈30% E1 conversion to estradiol (E2)), stopped with 60 µL 0.21 M ascorbic acid (#A92902, Sigma Aldrich, Taufkirchen, Germany) in methanol (#34966, Honeywell/Riedel-de Haen, Seelze, Germany):acetic acid (#1.00063.1000, Merck, Darmstadt, Germany) (99:1, *v*/*v*), and stored at 4 °C until steroid extraction.

Steroids were extracted from the reaction mixtures by solid-phase extraction (SPE) (Strata C18-E, 100 mg/mL; Phenomenex, Torrance, CA, USA, #8B-S001-EAK). SPE involved: column conditioning with 2 mL methanol, column equilibration with 2 mL water, sample loading, washing with 1 mL water, drying under vacuum (10 min) and elution with 0.4 mL methanol. Eluates were evaporated at 45 °C in a vacuum concentrator (Savant SPD 131 DDA-230, Thermo Fisher Scientific, Singapore) and reconstituted in 50% acetonitrile. Reverse-phase chromatographic separation of steroids (RP-HPLC) was performed on a C18 column (RP-HPLC ODS Hypersil C18, 250 × 4.6 mm; ThermoFisher Scientific, Singapore) using acetonitrile (Supelco, Singapore, #1.00029):water (Supelco, Singapore, #1.15333) (1:1, *v*/*v*) at 1 mL/min, 40 °C, and injection volume 10 µL. Radioactive steroids were detected with a Raytest Ramona 2000 radioflow detector (Elysia-Raytest, Straubenhardt, Germany) using Quickszint Flow 302 scintillation fluid. The assays were run in duplicate; three independent experiments were performed. Conversion was calculated as % conversion = (% E2/(% E2 + % E1) × 100, and the inhibition was calculated according to the % inhibition = [(% conversion of control − % conversion of sample)/% conversion of control] × 100. IC_50_ values were calculated in GraphPad Prism, version 10.0 (GraphPad Software, Inc., San Diego, CA, USA)using non-linear regression curve fitting with four parameters.

##### 17β-HSD1 Enzymatic Assay with Unlabeled Estrone

Enzymatic reactions (1 mL) were prepared in 2 mL tubes containing 100 mM sodium phosphate buffer (pH 6.5), NADPH (100 µM), E1 (70 nM), and 10 µL of test inhibitor in DMSO (or DMSO for controls; final DMSO concentration 1%). After mixing, 10 µL homogenate was added, and the reactions were incubated at 37 °C for 10 min in a thermoshaker. The reactions were stopped by adding 60 µL of 1.26 M ascorbic acid in methanol:acetic acid (99:1, *v*/*v*) and processed immediately by SPE.

Prior to SPE, each sample or calibrator (1 mL) was spiked with 10 µL of internal standard [2,3,4−^13^C_3_]-17β-estradiol (^13^C_3_-E2; Sigma-Aldrich, #719552) (final concentration 10 ng/mL) and incubated for 15 min. SPE was performed using Strata X polymeric reversed-phase columns (30 mg/mL; Phenomenex, #8B-S100-TAK) and involved column conditioning with 1 mL methanol, column equilibration with 1 mL water, sample loading, washing with 1 mL water, drying under vacuum (10 min), and elution with 1 mL methanol. The eluates were stored at −20 °C until LC-MS/MS analysis.

Chromatographic separation was performed on a Shimadzu Nexera XR system with a Kinetex XB-C18 column (100 × 4.6 mm; 2.6 µm; Phenomenex, #00D-4496-E0) with mobile phases A (5% methanol in water, 0.2 mM NH_4_F (Honeywell/Fluka, Charlotte, NC, USA, #52481)) and B (methanol, 0.2 mM NH_4_F). The gradient was as follows: 30–96% B (1–3 min), wash at 96% B (to 8 min), and re-equilibration (to 15 min); flow rate of 0.5 mL/min; and injection volume of 5 µL. Detection was performed on a Sciex 3500 triple quadrupole mass spectrometer (Framingham, MA, USA) in negative ESI mode. Quantification was based on analyte/internal standard peak area ratios using Analyst 1.6 software. The assays were run in duplicate; three independent experiments were performed. Conversion was calculated as % conversion = (% E2/(% E2 + % E1) × 100, and the inhibition was calculated according to the % inhibition = [(% conversion of control − % conversion of sample)/% conversion of control] × 100. IC50 values were calculated in GraphPad Prism, version 10.0 (GraphPad Software, Inc., San Diego, CA, USA) using non-linear regression curve fitting with four parameters.

#### 4.2.3. Inhibition Assay for 17β-HSD2 with ^3^H-labeled Estradiol

The bacterial homogenate of E. coli overexpressing 17β-HSD2 was previously prepared as described in [29] and stored in aliquots at −80 °C. Then, 20 µL homogenate was added to 300 µL reaction mixtures containing 100 mM sodium phosphate buffer (pH 7.5), cofactor NAD^+^ (380 µM) (Karl Roth, Karlsruhe, Germany, #AE11, substrate estradiol (E2) in DMSO (300 nM; Sigma Aldrich, Taufkirchen, Germany, #E2758), tritium-labeled E2 ([2,4,6,7−^3^H-E2]; Perkin Elmer, Singapore, #NET317250UC) (0.6 µCi/mL) and 10 µL of test inhibitor in DMSO (or DMSO for controls; final DMSO concentration 5%). The reactions were incubated in a water bath at 37 °C for 30 min (≈30% E2 conversion to E1), stopped with 60 µL 0.21 M ascorbic acid in methanol:acetic acid (99:1, *v*/*v*), and stored at 4 °C until steroid extraction. Steroid extraction with SPE and RP-HPLC was the same as for the 17β-HSD1 assay with ^3^H-labeled E1. Conversion was calculated as % conversion = (% E1/(% E1 + % E2) × 100, and the inhibition was calculated according to the % inhibition = [(% conversion of control − % conversion of sample)/% conversion of control] × 100. The assays were run in duplicate; three independent experiments were performed. IC50 values were calculated in GraphPad Prism, version 10.0 (GraphPad Software, Inc., San Diego, CA, USA) using non-linear regression curve fitting with four parameters.

### 4.3. Cell Proliferation Assays

The influence of ring D-substituted estrone derivatives on cell proliferation was evaluated using ovarian (Kuramochi, COV362, OVSAHO), endometrial (Ishikawa, HEC-1-A, KLE, RL95-2), and breast cancer (MCF-7) cell lines, together with non-malignant control cell lines (HIEEC, control for endometrial cancer, HIO80, control for ovarian cancer, MCF10A, control for breast cancer). Cell proliferation was assessed using the alamarBlue reagent (DAL1025, Thermo Fisher Scientific) according to the manufacturer’s instructions.

All information regarding the cell lines used in this article is already stored in databases. Database names: ATCC (American Type Culture Collection), ECACC (European Collection of Authenticated Cell Cultures), JCRB Cell Bank, and Cellosaurus (RRID resource). Accession numbers: MCF10A (ATCC CRL-10317; RRID:CVCL_0598), MCF7 (ATCC HTB-22; RRID:CVCL_0031), KLE (ATCC CRL-1622; RRID:CVCL_0374), HEC-1A (ATCC HTB-112; RRID:CVCL_0293), RL95-2 (ATCC CRL-1671; RRID:CVCL_0505), Ishikawa (ECACC 99040201; RRID:CVCL_2529), COV362 (ECACC 07071910; RRID:CVCL_2420), Kuramochi (JCRB0098; RRID:CVCL_1345), OVSAHO (JCRB1046; RRID:CVCL_3114), HIEEC (kindly obtained from Michael A. Fortier (Laval University, Quebec, Canada, on 4 April 2014 as p14), and HIO-80 (CVCL_E274; kindly obtained from Andrew K. Godwin (University of Kansas Medical Center, Kansas, USA on 20 October 2017 as p+72).

The cells were cultured under standard conditions (37 °C, humidified atmosphere with 5% CO_2_) in appropriate culture media without antibiotics and routinely tested negative for mycoplasma infection. Authentication of selected cell lines was confirmed by STR profiling.

Endometrial cancer cell lines Ishikawa and MCF-7 breast cancer cells were maintained in Eagle’s Minimum Essential Medium supplemented with 5–10% fetal bovine serum (FBS) and insulin where required. HEC-1-A cells were cultured in McCoy’s 5A medium with 10% FBS. KLE and RL95-2 cells were maintained in DMEM/F12 medium supplemented with 10% FBS, L-glutamine, and insulin (RL95-2 only).

Ovarian cancer cell lines Kuramochi and OVSAHO were cultured in RPMI medium supplemented with 10% FBS and L-glutamine, whereas COV362 cells were maintained in DMEM with 10% FBS and L-glutamine.

Control cell lines were cultured as follows: HIEEC in RPMI-1640 with 10% FBS and L-glutamine; HIO80 in a 1:1 mixture of Medium 199 and MCDB105 supplemented with 4% FBS and insulin; and MCF10A in DMEM/F12 supplemented with 5% FBS, insulin, EGF, and hydrocortisone.

The cells were used within the +8–+14 passage range and seeded in 96-well plates at densities ranging from 1 × 10^4^ to 8 × 10^4^ cells/mL, depending on the cell line.

Estrane compounds were dissolved in DMSO to prepare 0.01 M stock solutions and further diluted in culture medium. The final DMSO concentration did not exceed 1%. After 24 h of cell attachment, the cells were treated with increasing concentrations of estrane derivatives for 48 h. Subsequently, 20 µL of alamarBlue reagent was added and incubated for 4 h. Absorbance was measured at 570 nm with a reference wavelength of 600 nm using a BioTek microplate reader (BioTek Instruments, Inc., Winooski, VT, USA).

Background absorbance (compound without cells) was subtracted, and values from replicate wells (duplicate/triplicate) were averaged. Three independent experiments were performed. The results were normalized to untreated control cells. For compounds showing strong antiproliferative activity, half-maximal effective concentration (EC_50_) values were calculated from dose–response curves using GraphPad Prism (Version 10.0).

### 4.4. Fluorescent Confocal Microscopy

The intracellular localization of compound **4** was investigated on MCF-7 human breast adenocarcinoma cells by fluorescent confocal microscopy. The cells were seeded at a density of 1 × 10^5^ cells/well onto glass coverslips placed into 24-well plates. The following day, the cells were treated with 10 µM of compound **4** in complete culture medium for 2 and 4 h. After removing the compound **4**-containing medium, the cells were rinsed three times with PBS and subsequently fixed for 10 min in a pre-chilled methanol:acetone solution (1:1, *v*/*v*). Nuclear staining was performed using DAPI (1 µg/mL) for 10 min. After staining, the coverslips were mounted onto glass microscope slides with Fluoromount mounting medium (Merck, Darmstadt, Germany). Fluorescence imaging was carried out on an Olympus Fluoview FV10i confocal microscope (Olympus Corporation, Tokyo, Japan) equipped with a 60× objective lens. Excitation wavelengths of 550 nm and 405 nm were applied for compound **4** and DAPI, respectively, while fluorescence emission was collected at 580 nm for compound **4** and 461 nm for DAPI using 55% laser power. The acquired images were analyzed and processed with Olympus Fluoview Viewer software (version 3.1a).

### 4.5. Computational Simulations

Experimental structures of proteins were downloaded from the pdb database (pdb codes are 3C3U, 4XO7, 1EQU for AKR1C1, AKR1C2 and 17β-HSD1, respectively) [30,31,32], and the crystal structures were prepared using the protein preparation wizard from Maestro GUI of the Schrödinger Suite, applying normal physiological conditions. The AKR protein complexes with ligands **2** and **3** were generated by docking calculations applying the Glide package of the Schrödinger Suite (version 2025-4). The position of the glide box was determined by the original ligand pose according to the PDB structures with a 20 × 20 × 20 outer grid box. Among the different options, the extra-precision (XP) protocol was applied with enhanced ligand sampling. The predicted pose was selected according to the Glide Emodel scoring function by taking the complex with the best Emodel value, which provided the most plausible binding position. To determine the characteristic interactions in protein–ligand complexes, the Ligand Interaction Diagram was applied (version 2025-4).

Concerning the 17β-HSD1—**4** complex, the starting pose was prepared manually, where the sterane skeleton part was positioned in the binding pocket according to previous steroid docking results and the BODIPY part was oriented towards the solvent. Then, three independent molecular dynamics simulations of ligand **4** with the 17β-HSD1 enzyme were performed, resulting in 200 ns long trajectories using the SPC water model and the OPLS4 force field under physiological conditions (using a 0.15 M NaCl salt concentration and protonation state at pH 7.4). All the molecular dynamics calculations were performed with the Desmond package of the Schrödinger Suite Desmond Molecular Dynamics System, and each simulation was augmented with Simulation Interaction Diagram (SID) analysis (version 2025-4).

Experimental inhibition was quantified as inhibition percentages measured at a single ligand concentration of 100 µM, as shown in Equation (1). In the absence of full dose–response curves, inhibition percentages were converted into apparent dissociation constants (*K_d_*) by assuming a simple reversible 1:1 binding equilibrium between ligands and proteins after a direct correspondence between fractional inhibition and fractional target occupancy, as shown in Equations (2) and (3).

Under this assumption, the fraction of inhibited target (*I*) is approximated by the classical binding isotherm, as shown in Equation (4) [33,34]

(1) $$Inhibition\equiv I=\;\frac{\left[L\right]}{\left[L\right]+K_{d}}$$

where [*L*] is the ligand concentration and *K_d_* is the equilibrium dissociation constant. Rearranging the equation yields an estimate of *K_d_*:

(2) $$K_{d}=\left[L\right]\left(\frac{1-f}{f}\right)$$

where *f* is fractional inhibition:

(3) $$f\;\sim \;\;\frac{I}{100}$$

Binding free energies were subsequently estimated from the apparent dissociation constants using the standard thermodynamic relationship:

(4) $$\Delta G=RTlnK_{d}$$

## 5. Conclusions

This study reports the biological evaluation of 15β-substituted E1 derivatives as inhibitors of AKR1C1–3, 17β-HSD1, and 17β-HSD2. Compounds **2** and **3** were identified as selective AKR1C2 inhibitors with sub- to low-micromolar IC_50_ values, while compounds **1**–**3** displayed potent 17β-HSD1 inhibition in the low nanomolar range. Notably, compound **4** represents the first fluorescently labeled E1-based inhibitor of 17β-HSD1 that retains submicromolar inhibitory activity. The applied combination of enzymatic assays, cell viability studies, and molecular docking proved suitable for evaluating inhibitory potency and isoenzyme selectivity and for rationalizing the observed differences in ligand binding between highly homologous AKR1C isoforms. Computational results supported the experimentally observed AKR1C2 selectivity and highlighted the role of subtle active-site variations in determining ligand orientation and binding stability. Overall, this work introduces a versatile E1-based scaffold enabling potent and selective inhibition of steroid-metabolizing enzymes, together with the development of a functional fluorescent probe for 17β-HSD1. This dual functionality provides a valuable platform for both inhibitor optimization and intracellular mechanistic studies. Future studies should focus on improving isoenzyme selectivity, validating intracellular target engagement, and exploring the therapeutic potential of the most promising derivatives in advanced disease models.

## Figures and Tables

**Table 1:** Inhibition of the AKR1C1–3, 17β-HSD1 and 17β-HSD2 enzymes by the 15β-substituted estrone derivatives **1**–**5**.

Compd. Number			AKR1C1	AKR1C2	AKR1C3	17β-HSD1	17β-HSD2
**1**	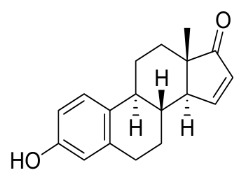	% inh. (µM)	25.00 (100)	25.00 (100)	NI (100)	98.26 (100)	73.65 (100)
		IC_50_ (CI)	(n.d.)	(n.d.)	(n.d.)	2.48 nM (1.22–4.24 nM)	(n.d.)
**2**	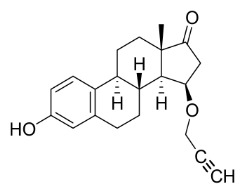	% inh. (µM)	32.50 (100)	99.1 (100)	56.20 (100)	100.00 (100)	41.35 (100)
		IC_50_ (CI)	(n.d)	0.67 (0.33–0.79)	(n.d)	1.97 nM (0.96–2.98 nM)	(n.d)
**3**	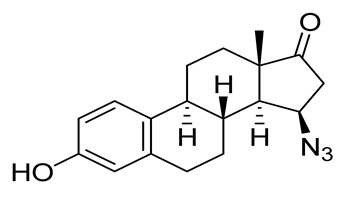	% inh. (µM)	62.50 (100)	97.50 (100)	NI (100)	100.00 (100)	71.75
		IC_50_ (CI)	(n.d.)	4.27 (1.65–7.90)	(n.d.)	4.02 nM (2.40–6.06 nM)	(n.d.)
**4**	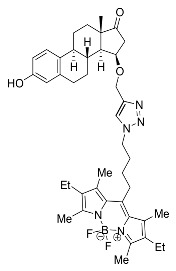	% inh. (µM)	20.00 (20)	20.00 (20)	0.00 (20)	98.09 (20)	34.83 (20)
		IC_50_ (CI)	(n.d.)	(n.d.)	(n.d.)	0.90 (0.42–1.36)	(n.d.)
**5**	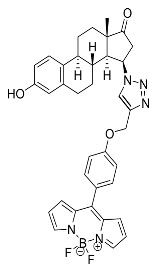	% inh. (µM)	20.00 (20)	0.00 (20)	12.50 (20)	23.88 (20)	37.83 (20)
		IC_50_ (CI)	(n.d.)	(n.d.)	(n.d.)	(n.d.)	(n.d.)
Positive control			93.40 (10)	94.20 (10)	95.00 (1)	90.77 (10)	NA

Positive control for AKR1C1, medroxyprogesterone acetate (10 µM); positive control for AKR1C2, ursodeoxycholic acid (10 µM); positive control for AKR1C3, medroxyprogesterone acetate (1 µM); positive control for 17β-HSD1, equilin (10 µM). IC_50_ values were calculated in GraphPad Prism using non-linear regression curve fitting with four parameters. IC_50_, half maximal inhibitory concentration; CI, confidence interval; NA, not available; NI, no inhibition; n.d., not determined; % inh.—% inhibition (tested conc. (µM)); (IC_50_, 95% CI (µM), unless otherwise stated).

**Table 2:** Cytotoxic effects of compounds (**1**–**5**) after 48 h treatment and EC_50_ values (μM) on various cancer cells and their respective controls.

Comp No.		Control	BC	Control	EC				Control	OC		
	[μM]	MCF10A	MCF7	HIEEC	KLE	HEC-1-A	ISHIKAWA	RL-95	HIO80	COV362	KURAMOCHI	OVSAHO
**1**	100	n.v.	n.v.	n.v.	n.v.	n.v.	n.v.	n.v.	n.v.	n.v.	n.v.	n.v.
	EC_50_	0.18	1.48	5.83	2.15	0.97	0.99	(n.d.)	0.58	4.54	5.87	(n.d.)
**2**	100	n.v.	12.92 ±12.97	73.67 ±27.21	29.49 ±11.97	n.v.	n.v.	42.46 ±1.64	n.v.	n.v.	39.27 ±22.76	<20% I
	EC_50_	(n.d.)	(n.d.)	(n.d.)	43.51	13.15	19.35	(n.d.)	(n.d.)	(n.d.)	(n.d.)	(n.d.)
**3**	100	n.v.	n.v.	n.v.	n.v.	31.18 ±2.97	n.v.	n.v.	n.v.	11.6 ±18.67	n.v.	n.v.
	EC_50_	0.22	1.42	6.93	2.12	(n.d.)	0.84	(n.d.)	0.83	(n.d.)	5.29	(n.d.)
**4**	100	44.14 ±3.85	30.64 ±0.08	<20% I	70.62 ±19.16	30.41 ±27.97	17.82 ±5.31	123.95 ±9.41	<20% I	29.19 ±25.43	120.64 ±14.86	<20% I
	EC_50_	(n.d.)	(n.d.)	(n.d.)	(n.d.)	(n.d.)	(n.d.)	(n.d.)	(n.d.)	(n.d.)	(n.d.)	(n.d.)
**5**	100	<20% I	<20% I	<20% I	<20% A	30.01 ±25.54	22.11 ±13.45	137.27 ±23.73	<20% A	47.55 ±2.94	<20% A	<20% I
	EC_50_	(n.d.)	(n.d.)	(n.d.)	(n.d.)	(n.d.)	(n.d.)	(n.d.)	(n.d.)	(n.d.)	(n.d.)	(n.d.)

% Cell viability in response to 100 µM estranes (mean +/− stdev) and EC_50_ values (μM), BC—breast cancer, EC—endometrial cancer, OC—ovarian cancer, n.v.—no viability, (n.d.)—not determined, <20% I—<20% inhibition, and <20% A—<20% activation. Values of inhibition and activation below 20% are excluded from the table. Values indicating less than 10% viability are considered to represent no viability.

**Table 3:** Docking scores and ΔG binding energies (in kcal/mol) of the investigated compounds in AKR1C1 and AKR1C2 enzymes.

Compd.	AKR1C1		AKR1C2	
	Calc. exp. ΔG binding energy (kcal/mol)	Docking score (kcal/mol)	Calc. exp. ΔG binding energy (kcal/mol)	Docking score (kcal/mol)
**2**	−5.0	−3.4	−8.2	−8.0
**3**	−5.8	−4.5	−6.6	−7.5

## Data Availability

Data are available upon request.

## Supplementary Materials

The following supporting information can be downloaded at https://www.mdpi.com/article/10.3390/cancers18121889/s1: Figure S1: Ligand 4 RMSF value during the three (run1, run2, run3) independent 200 ns long MD simulations. The two curves in the figures are the consequences of different fitting protocols of frames (protein fitting—brown line; ligand fitting—magenta line); Figure S2: Simulation Interaction Diagrams of the **4**–17β-HSD1 complex concerning the three independent simulations (run1, run2, run3). (green column: H-bond, purple column: hydrophobic interaction, blue column: salt bridge, red column: ionic interaction); Table S1: Experimental inhibition% and docking scores (kcal/mol) of compounds **2** (EM-1062) and **3** (EM-1082).
