# Supplementary material for: Targeting Steroid-Metabolizing Enzymes with 15β-Substituted Estrone Analogues: Dual Discovery of AKR1C2/17β-HSD1 Inhibitors and a Fluorescent 17β-HSD1 Ligand
Source: Cancers (Basel). 2026 Jun 10;18(12):1889. doi: 10.3390/cancers18121889 (PMC13296439; doi:10.3390/cancers18121889)
Supplement: Supplementary file 1 [file cancers-18-01889-s001.zip › cancers-4341293-supplementary.pdf]

cancers

MDPI

Article

Targeting Steroid-Metabolizing Enzymes with 15β-Substituted Estrone Analogues: Dual Discovery of AKR1C2/17β-HSD1 Inhibitors and a Fluorescent 17β-HSD1 Ligand

Vivien Resch [1,†], Marija Gjorgoska [2,†], Eva Hafner [2], Ildikó Bacsa [3], Benjamin Kovács [3], Tomaž Büdefeld [2,‡], Attila Hunyadi [3,4], Ildikó Huliák [5], Mónika Kiricsi [5], Gábor Paragi [1,6,7], Tea Lanišnik Rižner [2,*] and Erzsébet Mernyák [3,*]

[1] Department of Medicinal Chemistry, University of Szeged, Dóm tér 8, H-6720 Szeged, Hungary; resch.vivien@gmail.com (V.R.); paragi@sol.cc.u-szeged.hu (G.P.)
[2] Institute of Biochemistry and Molecular Genetics, Faculty of Medicine, University of Ljubljana, Vrazov trg 2, 1000 Ljubljana, Slovenia; marija.gjorgoska@mf.uni-lj.si (M.G.); eva.hafner@kobis.si (E.H.)
[3] Institute of Pharmacognosy, University of Szeged, Eötvös u. 6, H-6720 Szeged, Hungary; bacsa.ildiko@szte.hu (I.B.); kovacs.benjamin81@gmail.com (B.K.); hunyadi.attila@szte.hu (A.H.)
[4] HUN-REN-SZTE Biologically Active Natural Products Research Group, Eötvös u. 6, H-6720 Szeged, Hungary
[5] Department of Biochemistry and Molecular Biology, University of Szeged, Közép fasor 52, H-6726 Szeged, Hungary; huliak.ildiko@szte.hu (I.H.); kiricsim@bio.u-szeged.hu (M.K.)
[6] Institute of Physics, University of Pécs, H-7624 Pecs, Hungary
[7] Department of Theoretical Physics, University of Szeged, Tisza L. krt. 84-86, H-6720 Szeged, Hungary
* Correspondence: tea.lanisnik-rizner@mf.uni-lj.si (T.L.R.); mernyak.erzsebet@szte.hu (E.M.)
† These authors contributed equally to this work.
‡ Deceased author.

 https://doi.org/10.3390/cancers18121889

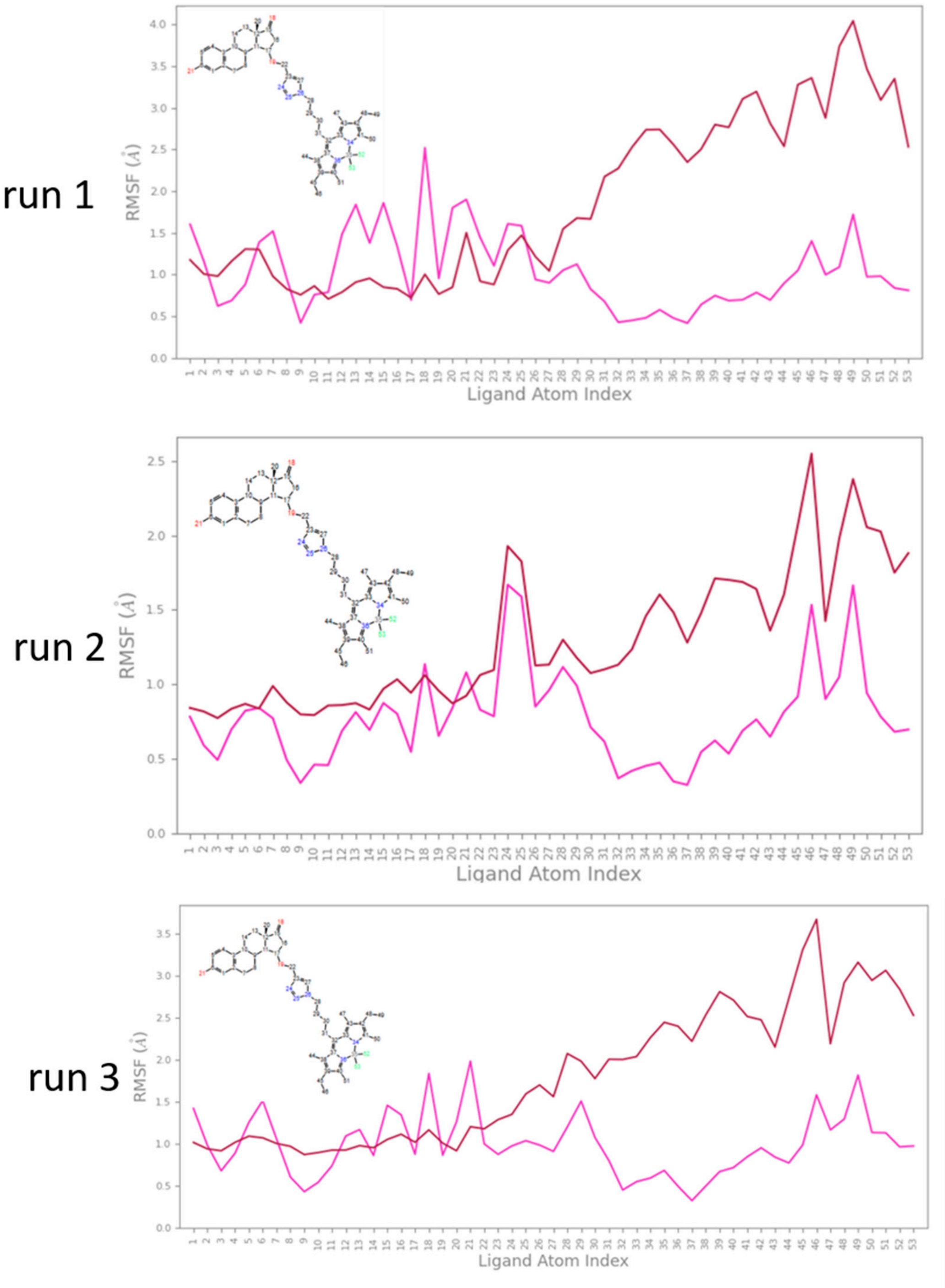

Figure S1. Ligand **4** RMSF value during the three (run1, run2, run3) independent 200ns long MD simulations. The two curves in the figures are the consequences of different fitting protocols of frames (protein fitting—brown line; ligand fitting—magenta line).

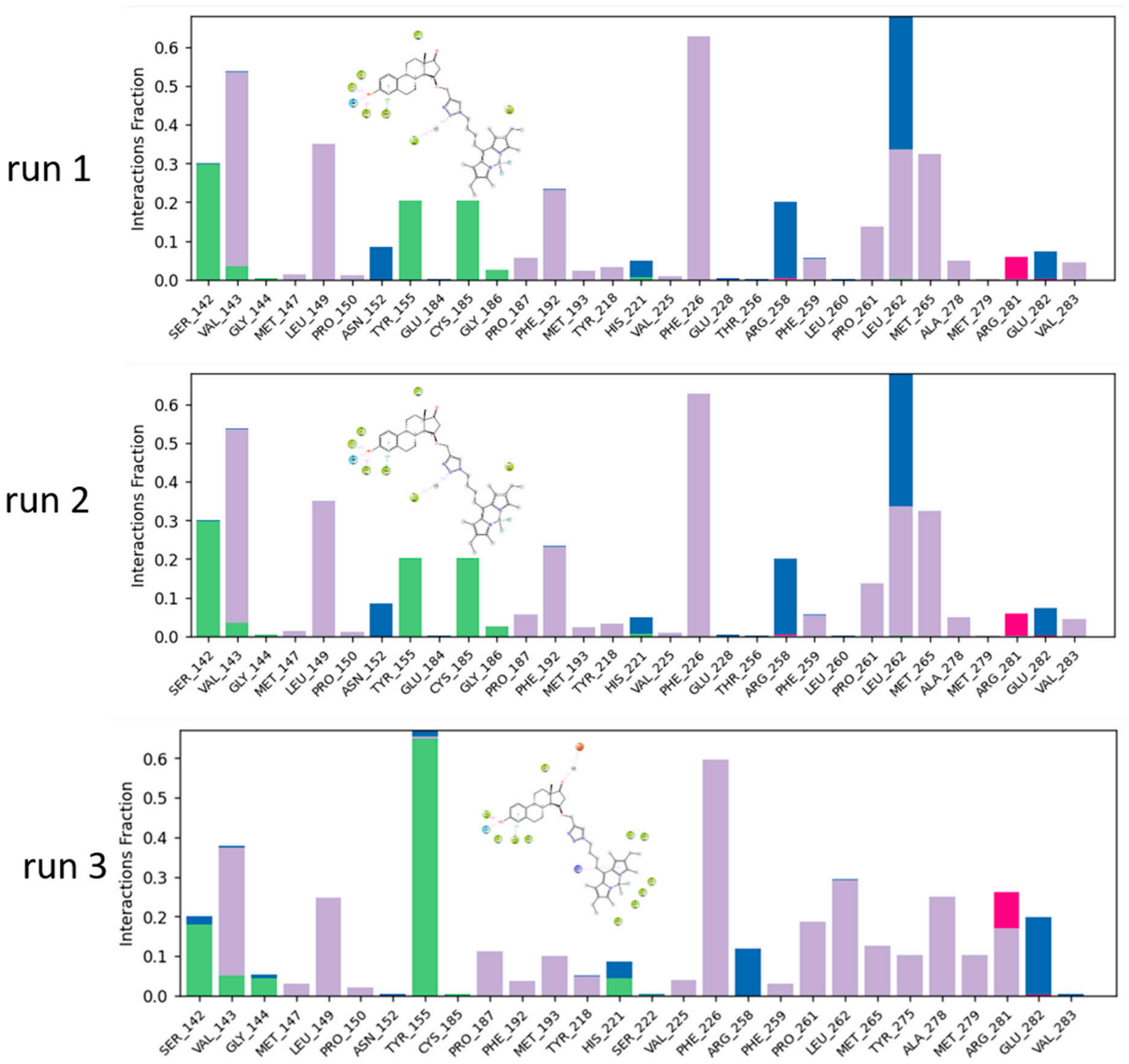

Figure S2. Simulation Interaction Diagrams of the **4**–17β-HSD1 complex concerning the three independent simulations (run1, run2, run3). (green column: H-bond, purple column: hydrophobic interaction, blue column: salt bridge, red column: ionic interaction)

Table S1. Experimental inhibition% and docking scores (kcal/mol) of compounds **2** (EM-1062) and **3** (EM-1082).

**Compd.**	**AKR1C1**		**AKR1C2**	
	Experimental inhibition% (100 μM)	Docking score (kcal/mol)	Experimental inhibition% (100 μM)	Docking score (kcal/mol)
**2**	33.0	-3.4	99.1	-8.0
**3**	62.5	-4.5	97.5	-7.5
